# Abnormal infant neurobehavior and later neurodevelopmental delays in children with critical CHD

**DOI:** 10.1017/S1047951122002013

**Published:** 2022-07-14

**Authors:** Kathleen Campbell, Lauren Malik, Courtney Jones, Zhining Ou, Angela Presson, Thomas A. Miller, Sarah Winter, Kristi Glotzbach

**Affiliations:** 1 Department of Pediatrics, Division of General Pediatrics, University of Utah, Salt Lake City, UT, USA;; 2 Department of Pediatrics, Division of Developmental and Behavioral Pediatrics, The Children’s Hospital of Philadelphia, Philadelphia, PA, USA;; 3 Department of Pediatrics, Intermountain Primary Children’s Hospital, Salt Lake City, UT, USA;; 4 Department of Internal Medicine, Division of Epidemiology, University of Utah, Salt Lake City, UT, USA;; 5 Department of Cardiovascular Services, Division of Pediatric Cardiology, Maine Medical Center, Portland, ME, USA; 6 Department of Pediatrics, Division of Critical Care Medicine, University of Utah, Salt Lake City, UT, USA

**Keywords:** Critical CHD, neurodevelopmental outcomes, infants

## Abstract

Infants with critical CHD have abnormal neurobehavior assessed by the Neonatal ICU Network Neurobehavioral Scales. This retrospective cohort study hypothesized associations between abnormal infant neurobehavior in the first month of life and later neurodevelopmental outcomes at 1–2 years of age. Associations between abnormal infant attention (orienting to and tracking stimuli) on the Neonatal ICU Network Neurobehavioral Scales and later motor, cognitive, and language neurodevelopmental outcomes on the Bayley Scales of Infant Development-III at follow-up were examined with descriptive statistics and univariable and multivariable regression. Multiple imputation was used to account for missing outcome data. 189 infants with critical CHD were included, and 69% had abnormal neurobehavioral attention scores. 58 (31%) returned as toddlers for neurodevelopmental follow-up, of which 23% had motor delay. Abnormal infant attention had high sensitivity (92%, 95% CI 60–100%) but low specificity (36%, 95% CI 23–52%) for later motor delay. Higher infant attention scores were associated with higher later motor scores in univariable analysis (coefficient 3.49, 95% CI 0.52,6.46, p = 0.025), but not in multivariable analyses. Neither cognitive nor language scores were associated with infant attention scores. Lower birth weight and male sex were significantly associated with lower motor scores in multivariable analysis (p = 0.048, 0.007). Although impaired infant attention is interdependent with other clinical and demographic risk factors, it may be a sensitive clinical marker of risk for later motor delay. In children with critical CHD, impaired infant attention may be capturing early signs of abnormal visual-motor neurodevelopment.

Children with critical CHD require surgical or catheter-based intervention in the first year of life and commonly have neurodevelopmental delays including motor, cognitive, and language delays. The most prevalent neurodevelopmental delay in toddlers with critical CHD is motor delay, which is diagnosed in 12–69% of toddlers.^1–3^ The American Heart Association and American Academy of Pediatrics recommend periodic neurodevelopmental follow-up for children with critical CHD to diagnose neurodevelopmental delays and refer children for intervention.^4^ The Cardiac Neurodevelopmental Outcomes Collaborative provided recommendations in 2020 for specific developmental exams to identify neurodevelopmental delays, with a more extensive schedule starting with a neurobehavioral exam before discharge and an abbreviated schedule with the first assessment of neurodevelopment at 6 months of age.^5^ Although clinical factors such as low birth weight, genetic disorders, and poor feeding have been associated with worse neurodevelopmental outcomes, there is not currently a clinical marker that could identify children with higher risk for closer follow-up with the more extensive schedule.^3,6,7^ Early assessment with a clinical marker that is sensitive to neurodevelopmental delay could help identify children for additional support, early interventions, or closer and more extensive follow-up.

Neonatal neurobehavioral exams are clinical exams appropriate for the first few weeks of life which can provide useful information about infant development, guide therapies, and predict neurodevelopmental outcomes. Infant neurobehavior can be assessed with the NICU Network Neurobehavioral Scales (NNNS) which has been validated in healthy and hospitalised infants. Normative scores for the NNNS domains exist for term and preterm infants in the first few weeks of life up to 48 weeks corrected age.^8^ Impaired performance on the NNNS is associated with motor delays in toddlers, and behaviour problems, school readiness, and lower Intelligence Quotient in pre-school-aged children with a history of substance exposure in utero or preterm birth.^9–13^ In infants with critical CHD, attention subscale scores on the NNNS appear to be the most consistently abnormal. The attention portion of the NNNS tests coordinated eye and head movements necessary for visual-motor orienting and tracking.^14,15^ It is unknown whether the abnormal attention scores in infants with critical CHD relate to later neurodevelopmental outcomes, except for the known association of pre-operative attention subscale scores with worse feeding outcomes.^14^ It is plausible that early impairments in the visual-motor skills required for the attention task would be related to the early motor delay commonly diagnosed in toddlers with critical CHD. However, no studies to date have examined this association. This study hypothesised that abnormal attention scores on the NNNS in infancy would be associated with later neurodevelopmental outcomes in toddlers with critical CHD and identify children at risk for motor delay.

## Methods

### Study design and patients

This was a single-centre, retrospective cohort study that included infants with critical CHD who had cardiac surgery with cardiopulmonary bypass at less than 30 days of age and had a post-operative NNNS evaluation between August 2015 and February 2020. Demographic and clinical information was collected on all infants with critical CHD in this time period and stored in a research database. Infants were excluded if they were premature (<37 weeks) or were diagnosed with a genetic disorder with known neurodevelopmental impairment (Trisomy 21 and 22q11.2 deletion). Neurodevelopmental follow-up between 1 and 2 years of age was voluntary but, all infants who met inclusion criteria were referred for outpatient neurodevelopmental evaluation. All eligible infants were included in the descriptive analysis to capture the effect of selection bias, but those not seen in follow-up clinic were not included in the primary outcome. Critical CHD lesions were categorised anatomically as 1 – single ventricle with arch obstruction, 2 – single ventricle without arch obstruction, 3 – two ventricle with arch obstruction, and 4 – two ventricle without arch obstruction. The Society of Thoracic Surgeons-European Association for Cardio-Thoracic Surgery (STAT) category was applied to characterise complexity of surgery with higher number indicating greater complexity.^16^ Family income was estimated in 2019 dollars using zip code in the medical record.^17^ Institutional Review Board approval was obtained and consent was waived for this study.

### Measures

#### NNNS:

The NNNS (version 1) is a standardised, objective exam of newborn behaviour developed for use in healthy as well as at-risk populations of newborns < 48 weeks gestational age. The NNNS development and methods have been previously described.^13,18,19^ The NNNS utilises a standardised behavioural evaluation to determine 12 domain scores plus a stress scale that describe an infant’s situational neurobehavior. We have previously described our clinical utilisation of the NNNS.^15^ We focused on the attention task (sometimes called the orienting task) of the NNNS because it was the most abnormal measure in the critical CHD population in previous studies,^14,15^ and deficits in a comparable domain have been described in critical CHD populations with other assessments.^20^ Briefly, licensed physical, speech, and occupational therapists certified in the NNNS assessment formally evaluate almost all (~90%) infants undergoing critical CHD surgery at our institution. The assessment is performed on all medically stable infants before cardiac surgery and just prior to hospital discharge when supplemental oxygen is at discharge dose and any potentially sedating medications are on an extended taper intended for continued long-term use at home or only available on an as-needed basis.

### NNNS attention task:

The infant must be in a quiet, alert state, and the examiner can use soothing techniques such as talking to, swaddling, and offering a pacifer to the infant to comfort them into this state. The examiner positions the infant in their lap and moves inanimate and animate stimuli (red ball, red rattle, face) through the infant’s visual field. They then rate whether the infant maintains a quiet, alert state and follows the stimulus with smooth eye and head movements. Scores range from 1 to 9 and the summary score is the mean of all attention items. A score of 3 or less generally means the child struggled to maintain a quiet alert state and focus visual attention on the stimulus long enough to follow movement. Scores of 4 indicate jerky eye movements but the infant can follow through 30 degrees of stimulus movement. Scores of 5 and higher indicate that the infant is generally smoothly following for at least 30 degrees with eyes and head.^19,21,22^ The mean attention summary scores of healthy, term newborns in the first 2 days of life is 5.97 with a standard deviation of 1.1.^21^ For categorical analysis, we defined an abnormal attention score as less than 4.87, 1 standard deviation below the typical mean.^21^

The post-operative NNNS attention subscale score was selected for analysis (and not the pre-operative score) because we were interested in assessing the neurobehavioral status of the infant in closest proximity to their subsequent neurodevelopmental follow-up taking into account the impact of the hospital course. Realising that the infant’s state may be impacted by their peri-operative and hospital course (i.e. opioid or benzodiazepine tapers, anti-epileptic medication, duration of hospitalisation), we included these medications and clinical factors in the NNNS analysis. We hypothesised that these medications and clinical exposures might affect the NNNS exam. Medications included oxycodone, clonidine, morphine, methadone, lorazepam, midazolam, levetiracetam, and phenobarbital.

### Bayley scales of infant development-III

The Bayley scales of infant development-III is a validated, objective examination of neurodevelopment with standard scores in motor, cognitive, and language development. Published norms are a standard score of 100 in each subscale and standard deviation of 15.^23^ We used a cut-off subscale score of 85 (−1 SD) as a marker of significant delay for categorical analysis. The Bayley scales of infant development-III has three sections, which are administered separately by a speech-language pathologist (language) and a physical or occupational therapist (motor and cognitive) at our institution.

### Statistical analysis

We summarised demographics and clinical outcomes of interest with mean and standard deviation or median and interquartile range and range for continuous variables, and counts and percentages for categorical variables. For group comparisons, we used descriptive statistics including Wilcoxon rank sum test, chisquared, or Fisher’s exact test. We calculated sensitivity and specificity of NNNS attention as a predictor of Bayley scales of infant development-III motor outcome using the categorical thresholds described above and reported point estimates and 95% confidence intervals.

To examine the association between the NNNS attention score and the Bayley scales of infant development-III, we fitted univariable regression models on each Bayley scales of infant development-III subscale (motor, cognitive, and language) with NNNS attention as a predictor, as well as other known or suspected covariates that influence neurodevelopmental outcomes (age at surgery, type of critical CHD, hospital length of stay, duration of mechanical ventilation, gestational age at birth, birth weight, STAT category, insurance, distance from hospital centre, family income, race and ethnicity, sex, receiving therapies at home after discharge, age at neurodevelopmental follow-up, age at NNNS exam, days prior to hospital discharge at NNNS exam). We then carried forward predictors that were significant at the p = 0.10 level to the multivariable regression models to test the adjusted contributions of NNNS attention to variance in Bayley scales of infant development-III scores.

To examine reasons for lack of follow-up, we fitted multivariable logistic regression models with the binary outcome of presence or absence of follow-up visit and included the following hypothesised predictors of follow-up completion: NNNS attention scores, lesion categories, birth weight, STAT category, insurance type, distance from referral centre (miles), age at NNNS (day of life), and deceased status. We applied Firth’s bias correction to the logistic regression due to unbalance event rates that all deaths occurred in the non-response group.^24^

We reported regression coefficients and odds ratios, and their 95% confidence intervals and p-values. We used the generalised variance inflation factor to assess multicollinearity among covariates in our multivariable model settings. Multicollinearity was considered tolerable if the generalised variance inflation factor was < 2.24, which is equivalent to variance inflation factor < 5.^25^ No variables were ultimately removed for inflation. Statistical significance was assessed at the 0.05 level. Statistical analyses were implemented using R version 4.0.3.^26^

### Statistical approach to data missingness

Considering the large number of subjects missing follow-up data (70%), we acknowledged that analysis of subjects who had complete data and ignoring information from incomplete data (complete case analysis) would be biased regardless of the reason why data on outcome were missing.^27^ Therefore, we implemented multiple imputation in addition under the assumption of missing at random, or missingness can be predicted from observed data.^28^ We simulated 70 completed observation data sets which matches the percentage of incomplete observations as recommended by White et al.^29^ Missing Bayley scales of infant development-III scores and missing values from other variables were simulated based on risk factors included in multivariable model and in addition, the counterpart Bayley scales of infant development-III scores.

Other than the approach mentioned above, we also considered using inverse probability weight to remove bias caused by non-response, in case of informative loss which is relative to the severity of illness or death to account for data missingness.^30^ The inverse probability weight creates a pseudo-population in which effect measure is equal to the effect measure had nobody been missing follow-up. However, based on findings from the data in which all deaths occurred in the non-response group, we suspected that death was a competing risk of loss-to-follow-up such that once a subject dies no other outcomes can occur.^31^ The competing risk violates the consistency condition of inverse probability weight analysis which requires the intervention must be well-defined. The effect measures may be relatively well-defined when loss-to-follow-up is the only form of censoring, versus death is also a form of censoring. We therefore withheld the inverse probability weight analysis and instead presented complete case analysis for reference purpose and results from multiple imputations as the primary analysis of our study.

## Results

### Participants characteristics

**Figure 1.**
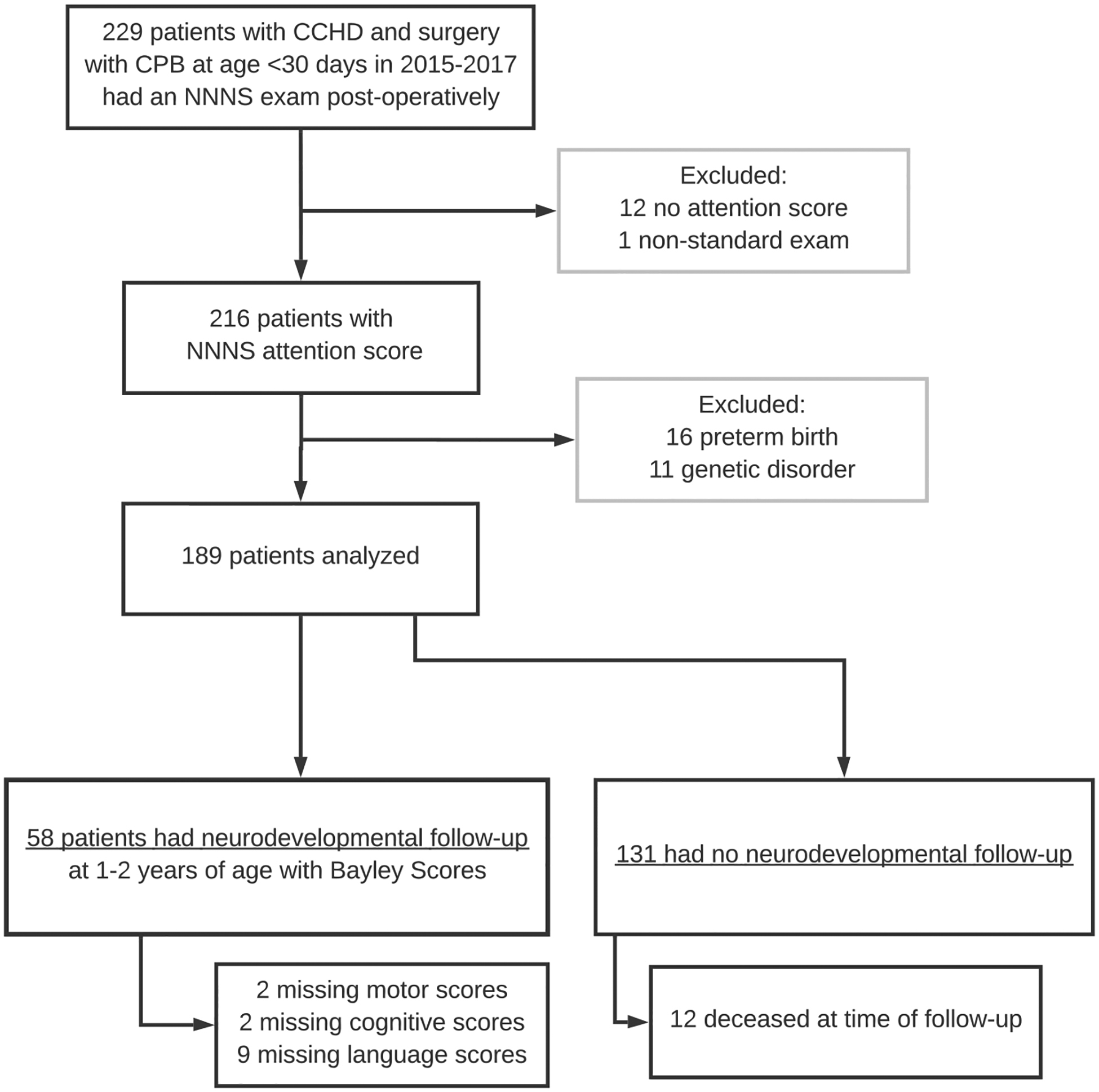
Subject selection and exclusion.

189 children met inclusion criteria and 58 of these had follow-up data for the primary outcome of Bayley scales of infant development-III scores at age 1–2 years (Fig 1). One infant was wearing a continuous positive airway pressure device during the NNNS, which is not standard practice, and they were excluded from the analysis. 11 infants did not have scorable NNNS attention scores due to not achieving a calm, regulated state during the exam and they were excluded from the analysis. Children who did not complete a subtest of the Bayley scales of infant development-III (due to therapist selection of a different test appropriate to the clinical situation) were included in the analysis with the scores of the subtests they did complete (2 motor missing, 2 cognitive missing, 9 language missing, Fig 1).

Demographic and clinical variables were compared in eligible infants between those with and without the primary neurodevelopmental outcome data at follow-up (Table 1). There were significant differences between the two groups. Subjects who followed up were more likely to have private insurance (p = 0.002), higher birth weight (3.3 kg (IQR 3.0–3.6) versus 3.1 kg (IQR 2.9–3.5), p = 0.043), and lower surgical complexity (p = 0.002), and be living at the time of follow-up (p = 0.019). In subjects with neurodevelopmental follow-up data, the median age at surgery was 6 days (IQR 4.0, 7.8), median hospital length of stay was 24 days (IQR 18.0–31.8) and duration of mechanical ventilation 6 days (IQR 5–7). These variables were not significantly different between cohorts.

In multivariable logistic regression analysis, STAT category and deceased status were significant predictors of no neurodevelopmental follow-up (Table 2). Children with STAT category 4 had higher odds of no neurodevelopmental follow-up compared to category 2 or 3 (OR 5.55, 95% CI 1.71,19.35, p = 0.004). Additionally, children who were deceased had higher odds of lack of follow-up (OR 12.11, 95% CI 1.42,1600.86, p = 0.017, wide range due to all deceased patients having membership in the no neurodevelopmental follow-up group).

### NICU Network Neurobehavioral Scales attention scores and association with neurodevelopmental outcomes

Across the entire cohort (n = 189), the mean NNNS attention score was 4.5 (SD 1.1), median 4.3 (IQR 3.9–5.1), and 69% had a score in the impaired range (less than 4.87). There was no difference in age at which the NNNS was performed in infants with impaired NNNS attention compared to those with typical NNNS attention (median 21.7 days versus 22.4 days, p = 0.43). Sedating or analgesic medications were given or available as needed on the day of NNNS exam in 40% of patients. NNNS attention score did not differ by use of medication. Median NNNS attention score was 4.3 in the group without medication versus 4.4 in the group with medication (p = 0.97).

**Figure 2.**
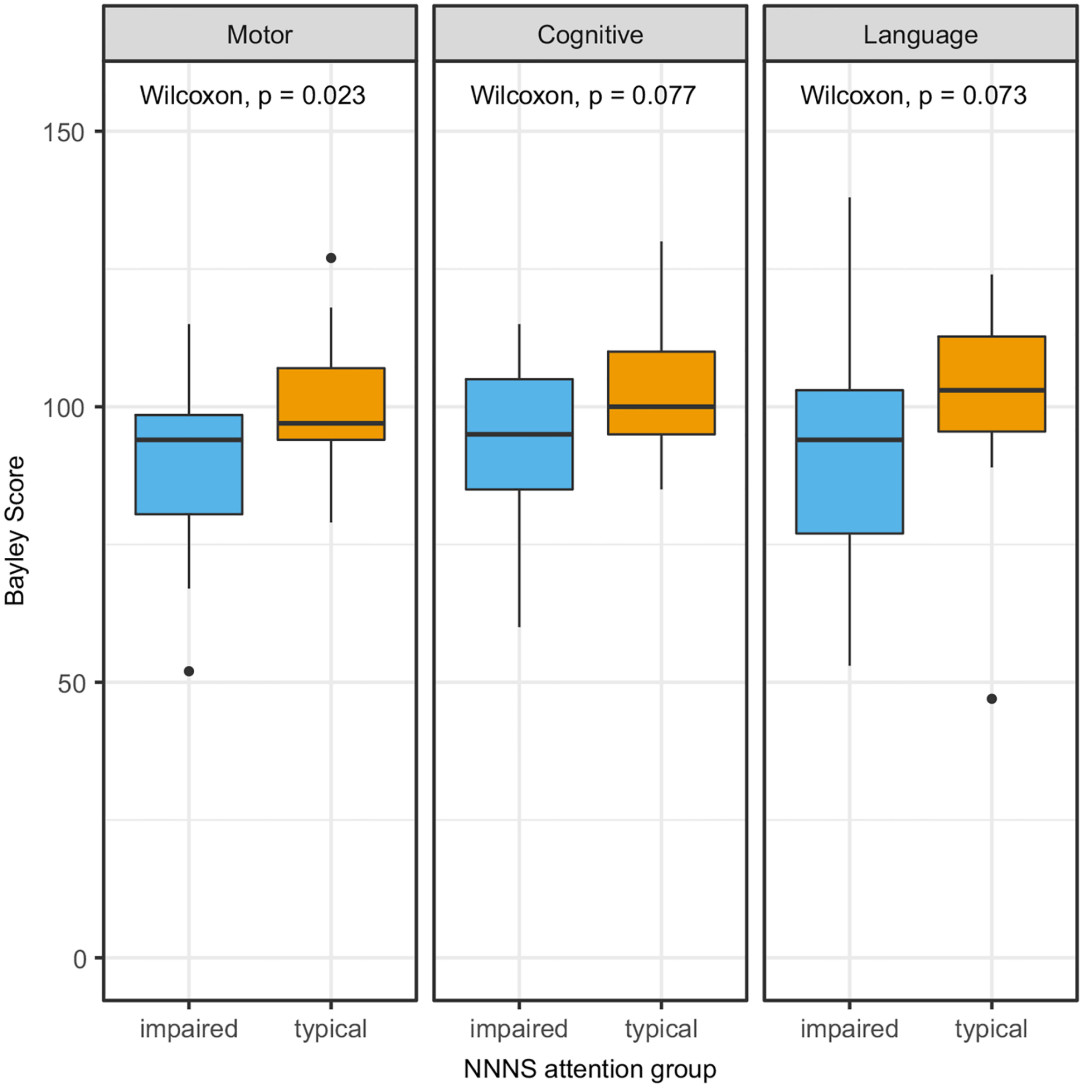
Boxplots of Bayley scores in impaired vs typical NNNS attention groups showing statistically significant difference in Motor scores but not Cognitive or Language scores.

In the group of 58 subjects with neurodevelopmental outcome data, the NNNS were administered at median age of 21 days of life (IQR 16–27) and 2.5 days (IQR 1.0–5.8) prior to hospital discharge (Table 1). The median NNNS attention score was 4.2 (IQR 3.7–5.1). Impaired NNNS attention had high sensitivity (92%, 95% CI 60–100%) but low specificity (36%, 95% CI 23–52%) for impairment on the Bayley scales of infant development-III motor scores. Children with impaired NNNS attention had significantly lower motor scores at follow-up (median 94 IQR 81–99 versus median 97 IQR 94–107, p = 0.023, overall range 52–127). Group differences were not statistically significant in Bayley scales of infant development-III cognitive scores (median 95 IQR 85–105 versus median 100 IQR 95–110, p = 0.077, overall range 60–130) and language scores (median 94 IQR 77–103 versus median 103 IQR 95–112, p = 0.073, overall range 47–138) (Fig 2).

In a univariable model with complete cases only, NNNS attention scores were positively associated with motor scores (coefficient 3.49, 95% CI 0.52–6.46, p = 0.025), but were not associated with cognitive or language scores (Table 3). Higher birth weight and higher family income were also associated with higher motor and cognitive scores. Shorter duration of mechanical ventilation, higher family income, and female sex were associated with higher language scores (Table 3). All other covariates were not associated with neurodevelopmental outcomes including age at surgery, lesion category (type of critical CHD), length of hospitalisation, gestational age at birth, STAT category, insurance, distance from hospital centre, race and ethnicity, receipt of developmental therapies after discharge, age at follow-up, age at NNNS, and days prior to hospital discharge at NNNS.

Results of the multivariable regression from multiple imputation analyses are presented in Table 4. When controlling for other potential predictors, NNNS attention was not associated with Bayley scales of infant development-III scores in any category. Higher Bayley scales of infant development-III motor scores were significantly associated with higher birth weight (coefficient 7.49, 95% CI 0.08, 14.91, p = 0.048), and female sex (coefficient for male −7.82, 95% CI −13.46, −2.18, p = 0.007). Bayley scales of infant development-III cognitive scores had no significant associations with any variable being considered in multivariable analysis. Higher Bayley scales of infant development-III language scores were significantly associated with fewer ventilation days (coefficient −2.16, 95% CI −3.34, −0.97, p < 0.001) and female sex (coefficient for male −13.19, 95% CI −20.53, −5.85, p < 0.001) after adjusting for other variables

## Discussion

In this retrospective cohort study of infants with critical CHD who underwent early cardiac surgery, we found that impaired visual attention in infancy was associated with lower motor scores at follow-up at 1–2 years of age in univariable but not multivariable analysis. The NNNS attention score appears to be a clinical marker for risk, associating with other peri-operative factors that are known to be associated with neurodevelopmental outcomes. The NNNS attention score did not predict Bayley scales of infant development-III scores beyond the explanation of variance by a combination of several clinical factors. Low birth weight and male sex predicted worse motor scores in multivariable analysis. Similar to our study, two previous large studies have found that lower birth weight is associated with lower motor score in children with critical CHD.^6,7^ There is however variability between studies, with a third study finding that feeding by mouth at follow-up was the best predictor of 1- to 3-year-old outcomes and birth weight was not significant and yet another study finding that lower weight and device-assisted feeding were both associated with lower Bayley scales of infant development-III scores at 6–12 months of age.^3,32^ We did not include feeding variables in our analysis, but inpatient feeding progress was related to higher pre-operative NNNS attention scores in a previous study at our centre.^14^ In our study, male sex was a prominent predictor of worse neurodevelopmental outcomes, but there is also variability in whether sex is a significant predictor in other studies. Female sex has predicted higher mental developmental index (a similar measure to the cognitive and language scores combined on the earlier Bayley scales of infant development-III), while sex was not a significant predictor of outcomes in other studies.^3,6^ With heterogeneity across models at different centres with different subject samples, a clinical marker with high sensitivity for future risk of motor delay, even if not an independent risk factor from other clinical course variables, could be an important contribution to the evaluation of children with critical CHD to prioritise follow-up and intervention. The NNNS attention task is a simple clinical test that in our sample had high sensitivity for motor delay.

Although children in the impaired infant NNNS attention group appeared to also have lower cognitive and language scores compared to infants with typical attention, lower cognitive and language scores were not significantly associated with impaired NNNS attention in univariable and multivariable analysis. In regression analysis, language outcomes were associated with longer duration of mechanical ventilation and male sex. Prior versions of the Bayley scales of infant development-III did not separate language scores from cognitive scores, and little has been written about predictors of language development in critical CHD at this young age. Longer duration of mechanical ventilation has been related to mental developmental index, which has now been broken up into the cognitive and language scales on the third edition of the Bayley scales of infant development.^7^ Another study found that language impairment at 12 and 24 months of age was associated with gestational age and birth weight in children with critical CHD, but this study did not include duration of mechanical ventilation as a potential predictor.^33^ Language scores have been related to attaining feeding by mouth by the time of follow-up.^3^ One study of motor skills, while inpatient, showed an association of worse motor skills with longer duration of mechanical ventilation and ICU length of stay.^34^ A previous study at our centre found that longer duration of mechanical ventilation was associated with delay in full oral feeds by discharge in children with critical CHD, including some children in this sample.^14^ Taken together, these studies indicate that longer duration of mechanical ventilation may delay oral-motor feeding and language skills, but we are not able to draw further conclusions about this relationship from this study. It is also possible that duration of mechanical ventilation is related to language development through other hospitalisation factors such as more time in the ICU, complications such as infections and reoperations, greater exposure to sedating medications, and longer time with reduced language exposure.

A clinically important majority of our sample (69%) showed impaired NNNS attention. We ruled out the possibility that this was related to age at NNNS administration, sedating or analgesic medications, prematurity, or genetic disorders that alter developmental trajectories by comparing NNNS attention scores between these groups and excluding subjects. One study of infants with very preterm birth (less than or equal to 32 weeks) showed similar NNNS attention scores at term equivalent age to our sample and found that impaired NNNS attention was related to lower fine motor scores at 4 years of age.^9^ Other studies of very preterm infants and substance-exposed infants had higher NNNS attention scores than our study.^35,36^ Nonetheless, lower NNNS attention has been shown to be associated with neonatal abstinence and lack of developmentally appropriate care while hospitalised. Developmental care practices (i.e. environmental modifications, non-pharmacologic soothing techniques, parental engagement) are increasingly used in hospitals for children with critical CHD. Continuing efforts to improve developmental care and the use of non-pharmacologic soothing measures during and after surgical recovery may be important to improve the neurobehavioral profile for infants with critical CHD.^36–38^ Further investigation is required to see if peri-operative developmental care practices or after discharge can alter the developmental trajectory in critical CHD.

This is the first study attempting to link an early marker of impaired neonatal neurobehavior to longer-term neurodevelopmental outcomes in critical CHD. More studies are needed that capture a larger proportion of the sample at the toddler follow-up to replicate and extend these findings. Estimates of the proportion of children captured for neurodevelopmental follow-up are often not presented and vary depending on age at follow-up and research enrollment versus complete clinical follow-up. Two of the single-center studies cited in this article provided capture data and reported 45% and 57%.^2,7^ In our cohort, neurodevelopmental follow-up capture was lower at 31%, even though 100% of the patients who had an NNNS evaluation had been referred for toddler neurodevelopmental follow-up. We attempted to account for these subjects with the multiple imputation analysis and analysed reasons for attrition. Death and higher complexity (although not the highest complexity) were significant independent predictors of loss-to-follow-up in multivariable analysis. There are multiple potential reasons for lack of follow-up that we considered but are difficult to measure without qualitative studies, including families of low complexity cases perhaps not seeing the importance of follow-up, and the highest complexity cases perhaps having competing needs for medical follow-up. Capturing a greater proportion of subjects for follow-up will be important to test these hypotheses but remains a challenge in many centres. In the largest multicenter study of 14 of the most highly resourced cardiac neurodevelopmental follow-up programmes, only 27% of eligible patients in the 11–30 month age range returned for neurodevelopmental follow-up, and disparities and limited resources to support complete follow-up were contributors to low follow-up.^39^ This highlights the clinical need for an early, inpatient marker of neurodevelopment such as the NNNS, with the potential to identify patients at highest risk of developmental delays, even if it is interdependent with clinical course, and target measures to ensure neurodevelopmental follow-up in critical CHD patients.

Our study has several limitations. The results of the primary outcome were likely impacted by selection bias due to the dependence on patient follow-up compliance. However, our follow-up rate is consistent with the national average for neurodevelopmental follow-up in critical CHD and so may be comparable to other studies of children with critical CHD in this age range. We attempted to control for this bias by including all infants with a Network Neurobehavioral Scales exam at our institution with the multiple imputation-based analysis for missingness. However, we did not account for any differences between infants who did not have an NNNS exam at our institution and our study population. Institutionally, it is rare that a newborn with critical CHD does not receive an NNNS exam prior to discharge with the exception of staffing availability on the weekends or death prior to hospital discharge. Therefore, there is likely a selection bias in our study wherein we did not study the sickest patients with critical CHD. Generalisability and replicability of abnormal infant attention on the NNNS in critical CHD is unknown, as only a few centres are using the NNNS to assess infant neurobehavior, but low attention has been seen in other post-operative critical CHD cohorts utilising other neurobehavior measures.^20^ Our use of the third edition of the Bayley (BSID-III) during the study period likely underestimated the frequency of motor delay, as has been shown in other studies of children with critical CHD.^40^ To this end, since the time of the data collection our centre has added a motor evaluation at 9 months of age with an increased follow-up rate that exceeds national averages.

## Conclusion

We found that NNNS attention impairment was common in infants with critical CHD and a sensitive predictor of later motor delay at 1–2 years of age. Infant neurobehavioral exams, namely the NNNS, may be a useful surrogate for clinical risk predictors or combined with other clinical predictors to guide early developmental recommendations and follow-up. Future studies are needed to understand whether impaired NNNS attention is related only to early motor delays or if it is associated with later neurodevelopmental challenges of attention, visual-spatial skills, or executive function skills.

## Figures and Tables

**Table 1.** Demographic and clinical variables for the subjects with and without neurodevelopmental follow-up data. Continuous variables are summarised with the median (interquartile range). Categorical variables are reported as frequencies and percentage of total.

	Neurodevelopmental follow-up (n = 58)	No Neurodevelopmental follow-up (n = 131)	p-Value
Demographic variables			
Sex: Female	27 (46.6%)	56 (42.7%)	0.63^c^
Male	31 (53.4%)	75 (57.3%)	–
Race: non-White	3 (5.2%)	9 (6.9%)	0.76^f^
White	55 (94.8%)	122 (93.1%)	–
Ethnicity: Hispanic/Latino	7 (12.1%)	17 (13.1%)	0.85^c^
Not Hispanic/Latino	51 (87.9%)	113 (86.9%)	–
Income by zip code	68,775 (56,281, 91,557)	69,800 (56,752, 81,920)	0.58^w^
Insurance: None, unavailable	1 (1.7%)	10 (7.6%)	**0.002** ^f^
Private	41 (70.7%)	57 (43.5%)	–
Public	16 (27.6%)	64 (48.9%)	–
Lives in same state as hospital	49 (84.5%)	97 (74%)	0.83^f^
Lives in other state	9 (15.5%)	34 (26%)	–
Distance from hospital (miles):	27.0 (18.2, 82.5)	39.5 (19.0, 184.0)	0.06^w^
Clinical variables			
Gestational age at birth (weeks)	38.0 (38.0, 39.0)	39.0 (38.0, 39.0)	0.60^w^
Birth weight (kg)	3.3 (3.0, 3.6)	3.1 (2.9, 3.5)	**0.043** ^w^
Age at surgery (days of life)	6.0 (4.0, 7.8)	6.0 (4.0, 7.0)	0.30^w^
Lesion category: 1	22 (37.9%)	33 (25.2%)	0.10^f^
2	6 (10.3%)	7 (5.3%)	–
3	13 (22.4%)	46 (35.1%)	–
4	17 (29.3%)	45 (34.4%)	–
STAT category: 2, 3	10 (17.2%)	8 (6.2%)	**0.002** ^c^
4	24 (41.4%)	87 (66.9%)	–
5	24 (41.4%)	35 (26.9%)	–
Length of hospitalisation (days)	24.0 (18.0, 31.8)	21.0 (16.5, 28.0)	0.15^w^
Ventilation days	6.0 (5.0, 7.0)	5.0 (4.0, 7.0)	0.33^w^
Therapies after discharge: no	14 (24.6%)	7 (38.9%)	0.24^c^
yes	43 (75.4%)	11 (61.1%)	–
Deceased status: alive	58 (100%)	119 (90.8%)	**0.019** ^f^
deceased	0 (0%)	12 (9.2%)	–
Age at follow-up (months)	21.6 (20.4, 22.8)	NA	–
NNNS exam variables			
Age at NNNS (days of life)	21.0 (16.0, 27.0)	19.0 (15.0, 25.0)	0.10^w^
Discharge time after NNNS (days)	2.5 (1.0, 5.8)	3.0 (1.0, 7.0)	0.24^w^
NNNS attention score	4.2 (3.7, 5.1)	4.3 (3.9, 5.2)	0.41^w^

Statistical tests:

w Exact Wilcoxon rank sum test,

f Fisher’s exact test,

c Chi-squared test

Abbreviations: NNNS = NICU Network Neurobehavioral Scales; STAT = Society of Thoracic Surgeons-European Association for Cardio-Thoracic Surgery (a classification of surgical complexity). Lesion Category key: 1 = single ventricle with arch obstruction, 2 = single ventricle without arch obstruction, 3 = two ventricle with arch obstruction 4 = two ventricle without arch obstruction. Number of missing values: Birth weight 0,1; STAT 0,1; Distance from hospital 0,1; Ethnicity 0,1; Therapies after discharge 1,113.

**Table 2.** Multivariable Firth’s logistic regression to determine odds of no neurodevelopmental follow-up based on clinical and demographic variables.

	Odds ratio (95% CI)	p-Value
NNNS attention score	1.15 (0.83,1.6)	0.41
Lesion category:1	–	ref
2	0.62 (0.08,3.59)	0.60
3	1.97 (0.27,13.11)	0.49
4	2.39 (0.3,17.01)	0.39
Birth weight	0.66 (0.29,1.46)	0.30
STAT category: 2,3	–	ref
4	5.55 (1.71,19.35)	**0.004**
5	4.75 (0.5,42.68)	0.17
Insurance: None, unavailable	–	ref
Private	0.41 (0.04,2.32)	0.33
Public	1.19 (0.11,7.11)	0.86
Income by zip code	1 (0.99,1.01)	0.35
Deceased status: alive	–	ref
Deceased	12.11 (1.42,1600.86)	**0.017**
Age at NNNS (days of life)	0.98 (0.94,1.02)	0.31

Abbreviations: NNNS = NICU Network Neurobehavioral Scales; STAT category = Society of Thoracic Surgeons-European Association for Cardio-Thoracic Surgery (a classification of surgical complexity). Lesion Category key: 1 = single ventricle with arch obstruction, 2 = single ventricle without arch obstruction, 3 = two ventricle with arch obstruction 4 = two ventricle without arch obstruction.

**Table 3.** Results of univariable linear regression models to investigate the association between NNNS attention score, demographic variables, and clinical variables as predictors of neurodevelopmental outcome scores

	Bayley Motor Score n = 56		Bayley Cognitive Score n = 56		Bayley Language Score n = 49	
	Coeff (95% CI)	p-value	Coeff (95% CI)	p-value	Coeff (95% CI)	p-value
NNNS attention score	3.49 (0.52,6.46)	**0.025**	2.60 (−0.60,5.80)	0.12	4.13 (−0.40,8.66)	0.08
Age at surgery	0.20 (−0.70,1.11)	0.66	−0.12 (−1.06,0.81)	0.80	−0.29 (−1.56,0.99)	0.66
Lesion category: 1	–	ref	–	ref	–	ref
2	2.76 (−10.34,15.85)	0.68	6.29 (−6.97,19.54)	0.36	15.46 (−9.61,40.53)	0.23
3	0.26 (−9.95,10.46)	0.96	−3.78 (−13.84, 6.29)	0.47	−4.85 (−20.13,10.44)	0.54
4	3.15 (−6.19,12.50)	0.51	5.45 (−4.18,15.09)	0.27	−2.34 (−16.03,11.36)	0.74
Length of hospitalisation (days)	−0.26 (−0.55,0.03)	0.09	−0.22 (−0.53,0.09)	0.16	−0.20 (−0.65,0.25)	0.38
Ventilation days	−0.67 (−1.99,0.66)	0.33	−1.07 (−2.43,0.29)	0.13	−3.32 (−5.13,−1.52)	**<0.001**
Gestational age at birth	2.75 (−0.94,6.44)	0.15	0.82 (−3.09,4.73)	0.68	0.94 (−4.92,6.79)	0.75
Birth weight	10.13 (1.87,18.38)	**0.020**	8.92 (0.29,17.55)	**0.048**	3.79 (−9.05,16.63)	0.57
STAT: 2,3	–	ref	–	ref	–	ref
4	1.10 (−9.95,12.15)	0.85	−0.48 (−12.03,11.06)	0.93	2.56 (−13.43,18.55)	0.76
5	−2.62 (−13.61, 8.36)	0.64	−2.85 (−14.33, 8.63)	0.63	1.75 (−14.10,17.60)	0.83
Insurance: none, unavailable	–	ref	–	ref	–	ref
Private	11.63 (−16.83,40.10)	0.43	2.12 (−27.41,31.66)	0.89	4.83 (−36.65,46.32)	0.82
Public	12.57 (−16.54,41.69)	0.40	−2.67 (−32.79,27.46)	0.86	3.08 (−39.51,45.67)	0.89
Lives in same state as hospital centre	–	ref	–	ref	–	ref
Lives in other state	3.17 (−7.00,13.34)	0.54	6.04 (−4.99,17.08)	0.29	13.66 (−1.51,28.83)	0.08
Miles from hospital centre	0.00 (−0.04,0.04)	0.84	0.00 (−0.04,0.05)	0.84	−0.06 (−0.12,0.00)	0.06
Income by zip code (x$1,000)	2.10 (0.23,3.98)	**0.032**	2.34 (0.41,4.27)	**0.021**	3.92 (1.20,6.64)	**0.007**
Race: non-White	–	ref	–	ref	–	ref
White	14.93 (−13.10,42.95)	0.30	−4.35 (−25.35,16.65)	0.69	10.62 (−13.34,34.58)	0.39
Ethnicity: Hispanic/Latino	–	ref	–	ref	–	ref
Not Hispanic/Latino	6.34 (−5.66,18.34)	0.31	3.70 (−8.88,16.28)	0.57	10.60 (−6.80,28.00)	0.24
Sex: Female	–	ref	–	ref	–	ref
Male	−6.52 (−13.83,0.79)	0.09	−4.53 (−12.24,3.19)	0.26	−13.76 (−24.68,−2.85)	**0.017**
Therapies after discharge: No	–	ref	–	ref	–	ref
Yes	−6.17 (−14.76,2.42)	0.17	−7.78 (−16.31,0.76)	0.08	−11.61 (−25.17,1.95)	0.10
Age at follow-up	2.44 (−13.27,18.14)	0.76	0.68 (−15.65,17.01)	0.94	17.30 (−9.02,43.62)	0.20
Age at NNNS (days of life)	−0.20 (−0.62,0.21)	0.35	−0.07 (−0.51,0.37)	0.75	−0.08 (−0.71,0.55)	0.80
Days prior to hospital discharge	−0.43 (−0.94,0.08)	0.10	−0.45 (−0.98,0.08)	0.10	−0.40 (−1.17,0.37)	0.31

Abbreviations: Coeff = Coefficient; NNNS = NICU Network Neurobehavioral Scales; STAT category = Society of Thoracic Surgeons-European Association for Cardio-Thoracic Surgery (a classification of surgical complexity). Lesion Category key: 1 = single ventricle with arch obstruction, 2 = single ventricle without arch obstruction, 3 = two ventricle with arch obstruction 4 = two ventricle without arch obstruction.

**Table 4.** Results of multivariable linear regression with multiple imputation to investigate the association between NNNS attention score and neurodevelopmental outcome scores while controlling for demographic and clinical variables. n = 189.

	Bayley Motor Score		Bayley Cognitive Score		Bayley Language Score	
	Coeff (95% CI)	p-value	Coeff (95% CI)	p-value	Coeff (95% CI)	p-value
NNNS attention score	2.22 (−0.28, 4.71)	0.08	1.77 (−1.22, 4.76)	0.24	2.22 (−1.1, 5.54)	0.19
Length of hospitalisation (days)	−0.12 (−0.25, 0.01)	0.07	–	–	–	–
Ventilation days	–	–	–	–	−2.16 (−3.34, −0.97)	**<0.001**
Birth weight	7.49 (0.08, 14.91)	**0.048**	3.93 (−4.33, 12.19)	0.34	–	–
Lives in same state as hospital	–	–	–	–	–	ref
Lives in other state	–	–	–	–	4.1 (−7.02, 15.22)	0.46
Miles from hospital	–	–	–	–	0 (0, 0.01)	0.26
Income by zip code (x$1,000)	1.36 (−0.16, 2.88)	0.08	1.81 (−0.05, 3.67)	0.06	2.18 (−0.39, 4.75)	0.09
Sex: Female	–	ref	–	–	–	ref
Male	−7.82 (−13.46, −2.18)	**0.007**	–	–	−13.19 (−20.53, −5.85)	**<0.001**
Therapies after discharge: No	–	–	–	ref	–	ref
Yes	–	–	−5.92 (−13.38, 1.54)	0.12	−6.67 (−17.37, 4.03)	0.21
Days prior to hospital discharge	–	–	−0.06 (−0.29, 0.17)	0.58	–	–

Abbreviations: Coeff = Coefficient; NNNS = NICU Network Neurobehavioral Scales; STAT category = Society of Thoracic Surgeons-European Association for Cardio-Thoracic Surgery (a classification of surgical complexity). Lesion Category key: 1 = single ventricle with arch obstruction, 2 = single ventricle without arch obstruction, 3 = two ventricle with arch obstruction 4 = two ventricle without arch obstruction.
